# Skin wound healing assessment via an optimized wound array model in miniature pigs

**DOI:** 10.1038/s41598-021-03855-y

**Published:** 2022-01-10

**Authors:** Ting-Yung Kuo, Chao-Cheng Huang, Shyh-Jou Shieh, Yu-Bin Wang, Ming-Jen Lin, Ming-Che Wu, Lynn L. H. Huang

**Affiliations:** 1 Department of Biotechnology and Bioindustry Sciences, College of Bioscience and Biotechnology, National Cheng Kung University, Tainan, Taiwan; 2 Livestock Research Institute, Council of Agriculture, Executive Yuan, Tainan, Taiwan; 3 Department of Pathology, Kaohsiung Chang Gung Memorial Hospital and Chang Gung University College of Medicine, Kaohsiung, Taiwan; 4 Biobank and Tissue Bank, Kaohsiung Chang Gung Memorial Hospital, Kaohsiung, Taiwan; 5 Division of Plastic and Reconstructive Surgery, Department of Surgery, National Cheng Kung University Hospital, College of Medicine, National Cheng Kung University, Tainan, Taiwan; 6 Institute of Clinical Medicine, College of Medicine, National Cheng Kung University, Tainan, Taiwan; 7 Research Center of Excellence in Regenerative Medicine, National Cheng Kung University, Tainan, Taiwan; 8 International Center for Wound Repair and Regeneration, National Cheng Kung University, Tainan, Taiwan

**Keywords:** Animal biotechnology, Biological models, Experimental models of disease

## Abstract

An appropriate animal wound model is urgently needed to assess wound dressings, cell therapies, and pharmaceutical agents. Minipig was selected owing to similarities with humans in body size, weight, and physiological status. Different wound sizes (0.07–100 cm^2^) were created at varying distances but fail to adequately distinguish the efficacy of various interventions. We aimed to resolve potential drawbacks by developing a systematic wound healing system. No significant variations in dorsal wound closure and contraction were observed within the thoracolumbar region between boundaries of both armpits and the paravertebral region above rib tips; therefore, Lanyu pigs appear suitable for constructing a reliable dorsal wound array. Blood flow signals interfered with inter-wound distances ˂ 4 cm; a distance > 4 cm is therefore recommended. Wound sizes ≥ 4 cm × 4 cm allowed optimal differentiation of interventions. Partial- (0.23 cm) and full-thickness (0.6 cm) wounds showed complete re-epithelialization on days 13 and 18 and strongest blood flow signals at days 4 and 11, respectively. Given histological and tensile strength assessments, tissue healing resembling normal skin was observed at least after 6 months. We established some golden standards for minimum wound size and distance between adjacent wounds for effectively differentiating interventions in considering 3R principles.

## Introduction

An ideal preclinical animal model is crucial for assessing wound healing and management to meet the growing demand for developing biomedical products^1,2^. Animal models simulating distinct human skin wound conditions have been developed to evaluate the effectiveness of different interventions^3–8^. Nevertheless, some drawbacks persist in existing models^9^. The loosened skin of small laboratory mammals, including rodents and rabbits, differs from human skin, closing the wounds via contraction. This characteristic, on the contrary, may benefit for evaluation of anti-contraction effects of biomaterials in wounds. Small animal selection is based on considerations such as laboratory space and cost^4–8^; however, these animals afford limited skin surface area, necessitating numerous animals to acquire sufficient data^10,11^, leading to errors and inconsistencies in the analyses of results.

**Figure 1:**
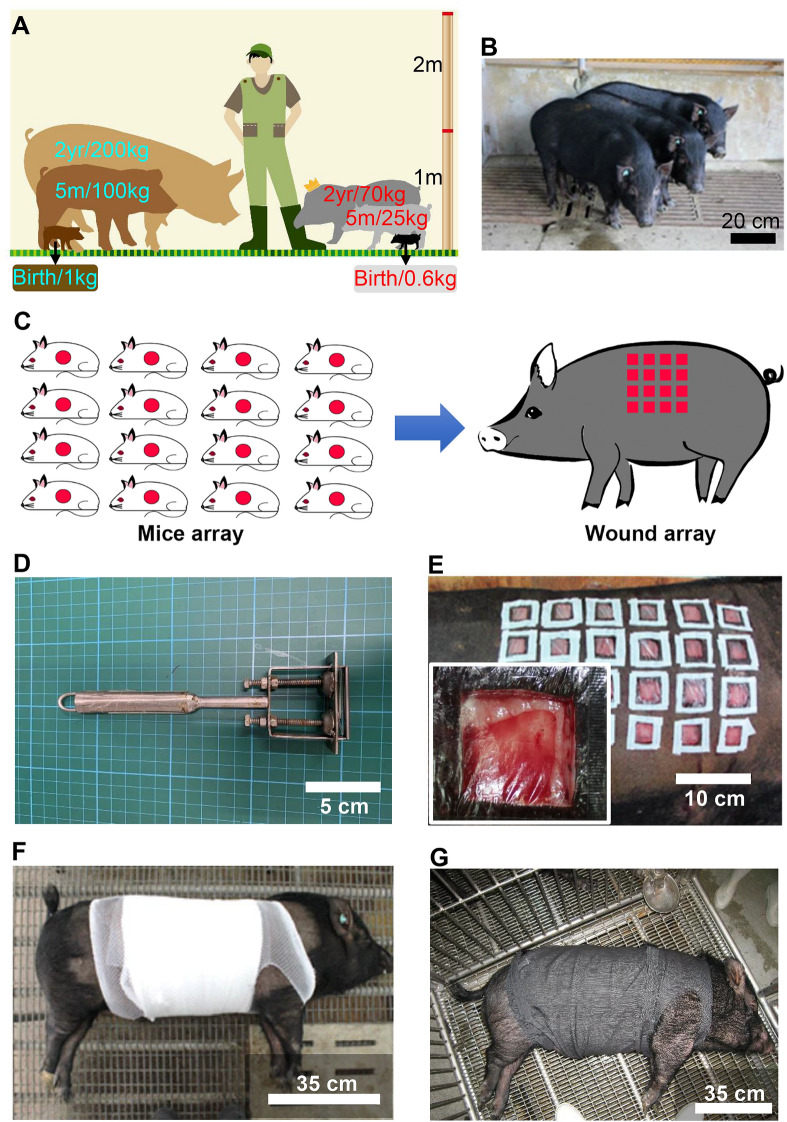
Wound array model of the Lanyu pig (*Susscrofa*). (**A**) A diagram comparing relative body sizes and weights of domestic Landrace pigs and Lanyu pigs. (**B**) Lanyu pigs with sexual maturity at 5 months of age have body weights of approximately 25 kg (bar = 20 cm). (**C**) Wound array on the dorsal skin of one pig is better than wounds created using numerous mice. (**D**) A handmade dermatome for wound creation is consisted of a grab handle, a blade in the end-plate and two long screws for adjusting wound depth. (**E**) As in the inserted picture, each wound should be initially covered with a polyurethane film (Tegaderm). Each wound should be further surrounded by adhesive plaster on the top of the polyurethane film (bar = 10 cm). (**F**) Wounds were further protected with sterile gauze, elastic bandage, and mesh bandage (bar = 35 cm). (**G**) A cloth further covered each pig for final protection.

Although sheep, goats, dogs, and other animals were also selected for external traumatic wound studies, they are more suitable for studying aspects such as bone, ligaments, and tendons. In contrast, pigs are better for wound studies due to larger dorsal surface area. Recently, pigs have been widely employed as a model for biomedical investigations because of their tight skin system that resembles that of humans, with a thick epidermis, distinct rete pegs, dense collagen fibrils, elastic fibrils in the dermis, and accessory hair and sweat glands. Both humans and pigs also demonstrate an identical wound healing mechanism, including restoration of the dermis, skin re-epithelialization, and skin contraction^12–14^. Furthermore, the body size of pigs is sufficiently large to provide an adequate skin surface to create multiple wounds on the same animal, thereby improving the reliability of results by eliminating extraneous factors associated with different animals^15,16^. In the last decade, commercially bred pig varieties such as Duroc, Yorkshire, and Landrace domestic pigs^15–17^ have been employed in skin wound healing studies. Despite the clinical validity and applicability of porcine models, larger breeding spaces and extensive resources are required to host these animals. Thus, miniature pigs, including Yucatan^18^, Gottinger^19^, and Lanyu^20^, have recently gained momentum as alternatives for wound healing studies, given the absence of the above limitations. Figure 1A shows the body weights of domestic Landrace pigs (*Sus scrofa domesticus*), Lanyu pigs (*Susscrofa*), and humans (*Homo sapiens*). Lanyu pigs reach sexual maturity of approximately 25 kg at 5 months of age, and their body weights are approximately 70 kg at 2 years of age. Conversely, domestic Landrace pigs reach sexual maturity at 5 months of age with a body weight of approximately 100 kg, and the body weight is approximately 200 kg at 2 years of age. Therefore, the body weights of Lanyu pigs were closer to the body weights of humans (Fig. 1A). Our previous study^20^, performed using a miniature Lanyu pig, revealed that the body weight and skin wound healing features of this breed resembled those of humans.

Almost all wound studies are performed on the dorsal surface, and wound sizes ranging between 0.07 and 100 cm^2^ can be established for different purposes. Elgharably et al.^21^ have reported 64 wounds in each domestic white pig, with a diameter of 3 mm and a depth of 7 mm, to evaluate multiple healing mechanisms associated with a modified collagen gel. Shevchenko et al.^22^ have punched 24 wounds in each female large white pig (wound size of 8 mm) to assess the integration of various material formulations. Agren et al.^23^ have punched 16 wounds in each domestic female white pig (wound size of 10 mm) to examine the efficacy of collagenase. These small punch wounds accelerate the healing process and achieve the purpose of analyzing various molecules. However, the healing process was extremely rapid to distinguish the effectiveness of various treatments. Velander et al.^24^ and Vamvanij et al.^25^ have created 18–21 wounds in each pig, with wound sizes measuring 1.5 cm × 1.5 cm. Furthermore, some researchers have established multiple excisional wounds in each pig, using wound sizes measuring 2 cm × 2 cm^26^, 2.5 cm × 2.5 cm^27^, 3 cm × 3 cm^28^, 4 cm × 4 cm^29,30^, or 5 cm × 5 cm^31^. Velander et al.^24^ have observed delayed wound healing in diabetic pigs exhibiting glucose levels > 500 mg/dL. Most studies failed to distinguish the efficacy of various interventions owing to the lack of proper wound sizes. Occasionally, wound expansion^30^ was observed due to skin growth when skin injury was established in a young pig. This phenomenon further results in a parabola in the wound contraction curve, deviating from a normal curve that concaves downward when no skin expansion exists^32^. Moreover, although wound distances of 2 cm^33^, 2.5 cm^26^, and 3–5 cm^34^ were mentioned in some studies, none investigated their effects on the healing process.

On reviewing accumulated evidence and considering the above-listed potential drawbacks, the present investigation was undertaken to perform a systematic wound study on the dorsal skin of mature Lanyu minipigs at the thoracolumbar region between the boundaries of both armpits and the paravertebral region above the rib tips. Herein, we established the golden standards of optimal wound size and the minimum distance between adjacent wounds to effectively differentiate various interventions. Based on the above criteria, an optimized wound array model could provide a high-throughput platform for precisely screening the effectiveness of different wound dressings, the therapeutic role of cells, and the efficacy of pharmaceutical agents, while simultaneously examining the underlying histological and molecular mechanisms.

## Results

### Suitability of the Lanyu pig as a wound array model

As shown in Fig. 1B, a 5-month-old Lanyu pig with a body weight of approximately 25 kg possesses a smaller size, which is easier to handle and occupies a smaller feeding space. For regular wound studies, multiple mice (minimum six) are required to eliminate individual differences. Conversely, a pig with multiple wounds in an array, abbreviated as a wound array, could achieve the same purpose (Fig. 1C). This wound array model established using pig skin meets the 3Rs principles of reduction, replacement, and refinement for animal studies. After creating multiple wounds, each wound was covered with Tegaderm and adhesive for fixing the dressing (Fig. 1E). The full wound array area was covered using sterile gauze, elastic bandage, mesh bandage, and cloth for protection against wound trauma after surgery (Fig. 1F,G).

### Histological and structural consistencies in different dorsal skin regions

**Figure 2:**
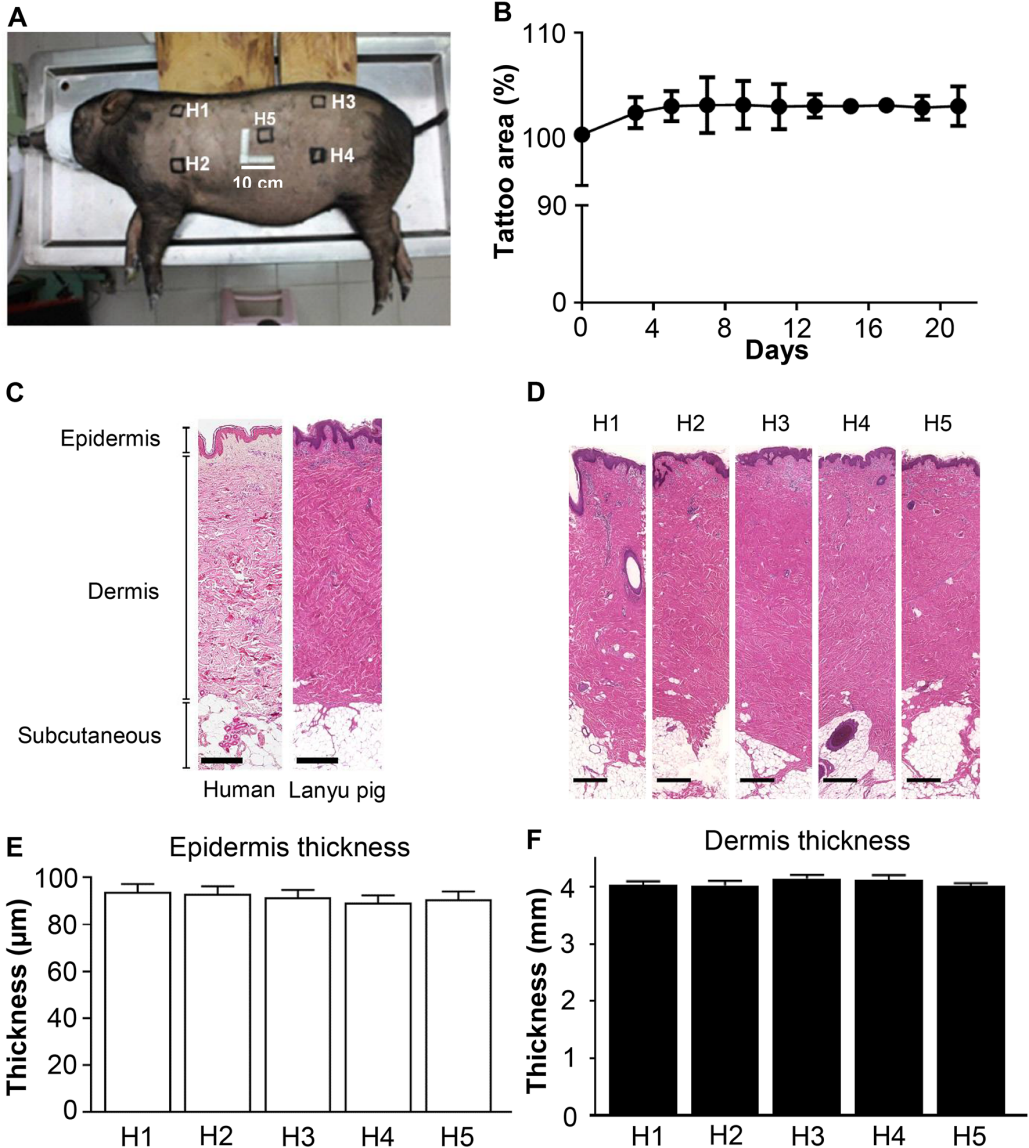
Variations in structure and histology at different regions of dorsal skin were compared on a Lanyu pig. (**A**) Square tattoos sized 2.5 cm × 2.5 cm were made on the dorsal skin of two Lanyu pigs. Five tattoos were indicated as H1, H2, H3, H4, and H5 (bar = 10 cm). (**B**) The size changes in each tattoo area were monitored for 22 days (*n* = 5). (**C**) Histological comparison of human skin and Lanyu pig skin (bar = 500 µm). (**D**) Histological comparison of skin at the five regions as indicated in (**A**) (bar = 500 µm). (**E**) The epidermal thicknesses of five tattoos were measured; data values are presented as mean ± standard deviation (SD). (**F**) The dermal thicknesses of the five tattoos were measured; data values are presented as mean ± SD. No significant differences in epidermal and dermal thickness were observed among tattoo groups using ANOVA statistical analysis.

Lanyu pigs weigh approximately 25 kg on reaching sexual maturity at 5 months of age and approximately 70 kg at 2 years of age, thus resembling the average human body weight. To create an optimal pig skin wound array model, mature pigs were selected, and variations in skin structure and histology were assessed in five regions, H1, H2, H3, H4, and H5 (Fig. 2A), evaluated from the array center and boundaries. Twenty-two days post-surgery, no significant changes were observed in each tattoo area (2.5 × 2.5 cm) (Fig. 2B). The approximate 2% increase in tattoo areas on day 22 corresponded to skin expansion and average dorsal surface growth (0.1% per day). Histological examinations of human and Lanyu pig skin revealed similar epidermal, dermal, and subcutaneous layer distributions (Fig. 2C). There was no obvious variation in structure and histology among above-mentioned five regions (Fig. 2D). Differences in epidermal and dermal thickness were insignificant in these five tattoo regions (P > 0.05) (Fig. 2E,F). These findings confirmed that the dorsal skin of Lanyu pigs is suitable for constructing a reliable wound array.

### Consistency of wound closure and contractility of open wounds at various dorsal skin sites

**Figure 3:**
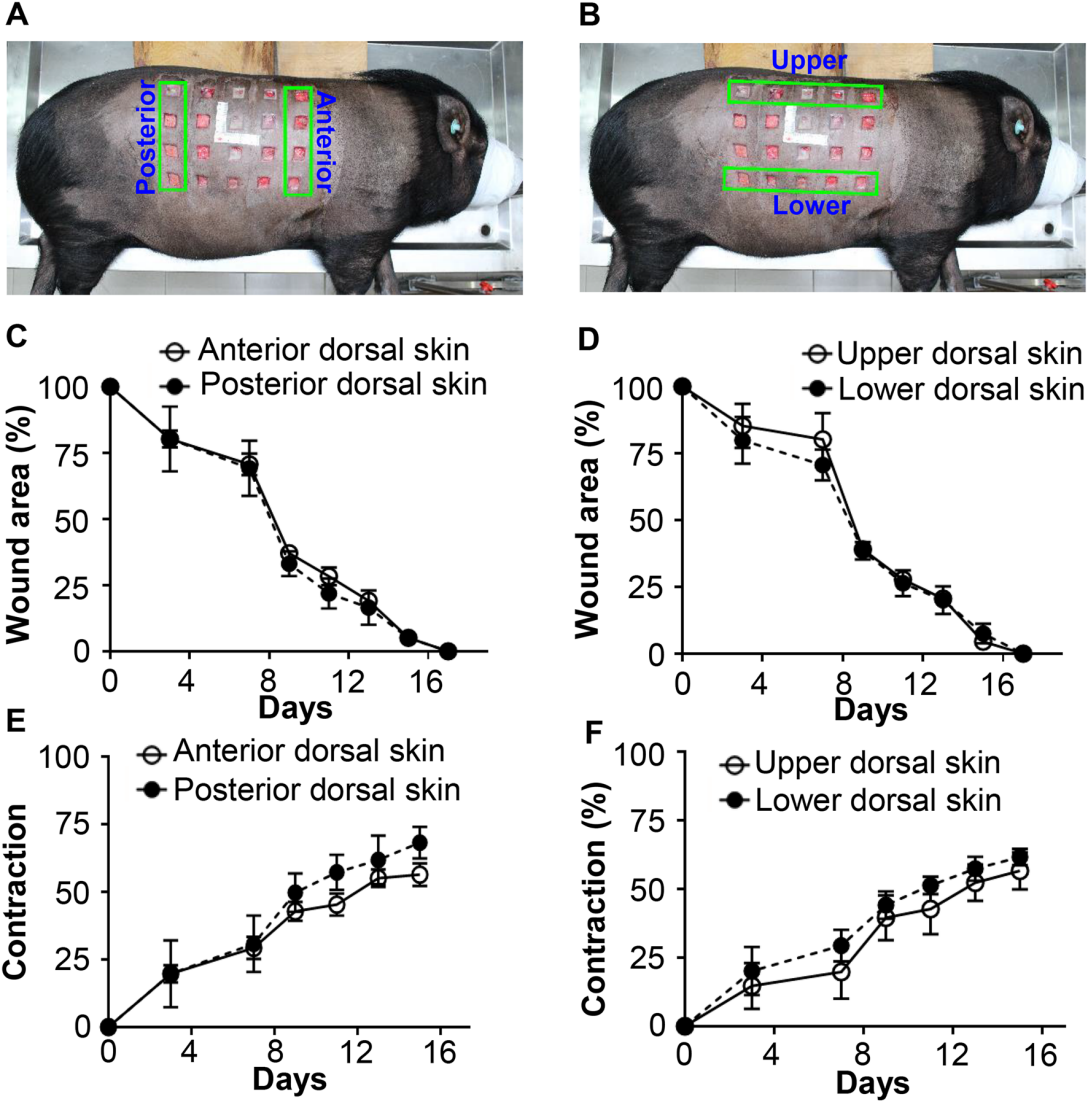
Variations in open wound closure and contraction, based on different regions of the dorsal skin. A wound array of 20 full-thickness wounds (2.5 cm × 2.5 cm × 0.6 cm) was created at different regions of dorsal skin as indicated in (**A**,**B**). (**A**) Photo presentation of wound regions at the anterior and posterior dorsal skin of a Lanyu pig. (**B**) Photo presentation of wound regions at the upper and lower dorsal skin of a Lanyu pig. (**C**,**D**) are changes in wound areas at different regions along with time. Data are presented as the percentage of wound area when compared with original wound sizes. (**E**,**F**) are percentage changes of wound contraction at different regions with time when compared with the original wound sizes. No significant differences between two groups were analyzed using student t-test statistical analysis.

To evaluate wounds created on the dorsum of Lanyu pigs at the boundaries of both armpits and above the rib tips, we compared wound closure and its contraction at the anterior and posterior regions (Fig. 3A,C,E) and those at the upper versus lower regions (Fig. 3B,D,F), respectively. Wounds measuring 2.5 cm × 2.5 cm × 0.6 cm in size closed the fastest during days 7–9, which was associated with the period of the strongest wound contraction, both for wounds in anterior and posterior regions (Fig. 3C,E) and the upper and lower regions (Fig. 3D,F). Regardless of location, no significant differences were observed in wound closure (P > 0.05) (Fig. 3C,D). There were no statistically significant differences in wound contraction at the different sites (P > 0.05) (Fig. 3E,F). Thus, the wound location on the dorsal skin did not significantly affect wound closure or contraction.

### Requirement of ≥ 4 cm interval distance between adjacent wounds to prevent interference during wound healing

**Figure 4:**
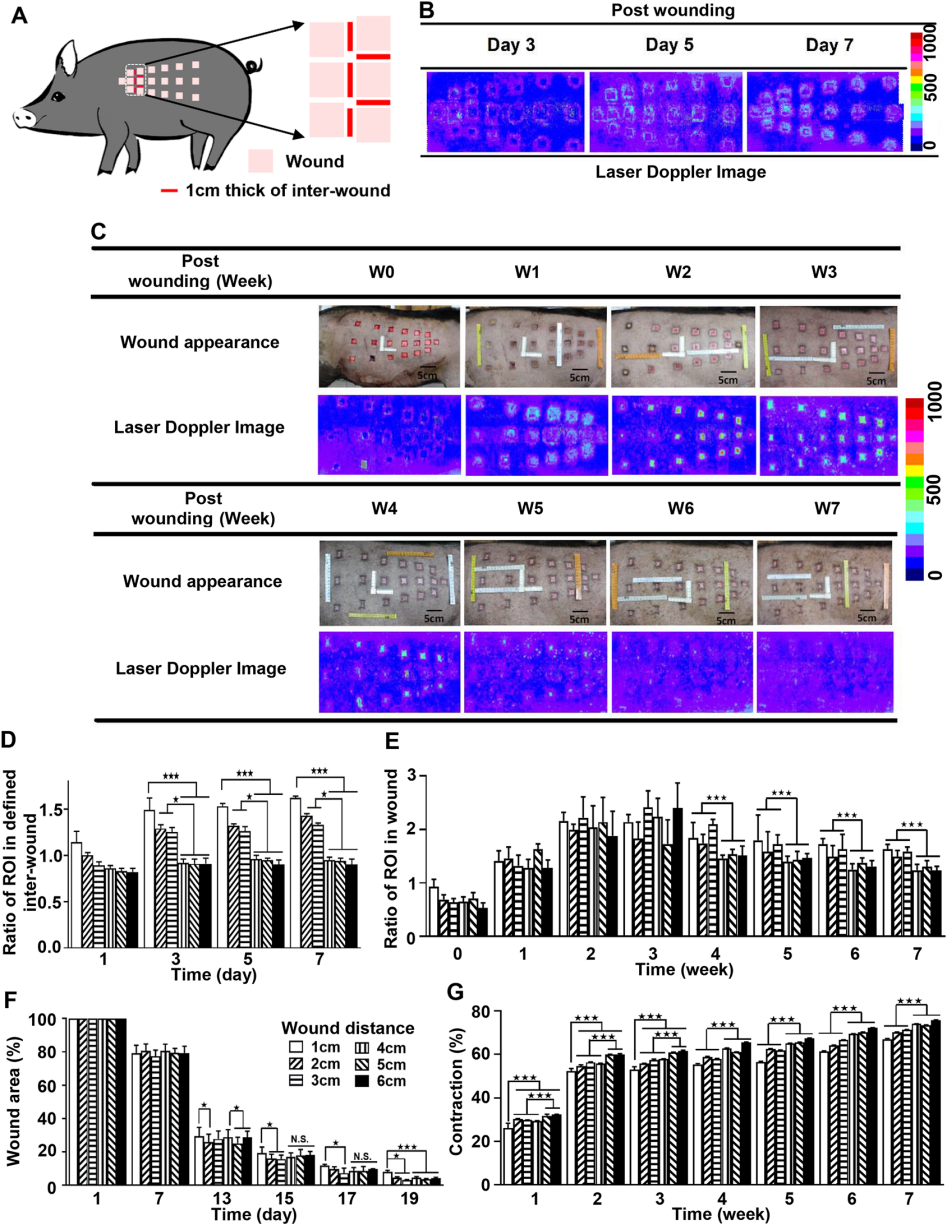
Studies to determine optimal distance between adjacent wounds sized 2.5 cm × 2.5 cm × 0.6 cm. Inter-wound distances of 1, 2, 3, 4, 5, and 6 cm were created for full-thickness wounds on the dorsal skin of two Lanyu pigs. (**A**) Schematic illustration indicates skin wounds created on a Lanyu pig with inter-wound distances of 1–6 cm from anterior skin to posterior skin. The opposite arrangement was established on the other pig. The red line indicates 1 cm-thick area from the midline of the inter-wound. (**B**) The laser Doppler images of the dorsal skin with various distances between adjacent wounds were determined on days 3, 5, and 7 postoperatively. (**C**) The appearance and laser Doppler images of the dorsal skin with wounds included were taken at weeks 0–7 postoperatively. (**D**) The ratios of ROI of various inter-wound distances at day 0, 3, 5, 7 were measured in comparison to the ROI of respective normal skin and data are expressed as mean ± SD (*n* = 10 for 2–6 cm of distances and *n* = 6 for 1 cm of distance). (**E**) The ratios of ROI of wounds with various distances of adjacent wounds were measured as indicated (*n* ≥ 6). (**F**) The closure of wounds with various distances of adjacent wounds was measured as the percentage of wound area when compared with each original wound size (*n* ≥ 6). (**G**) The percentages of wound contraction with various distances of adjacent wounds as indicated were measured with time postoperatively (*n* ≥ 6). Statistical significances of each parameter among various inter-wound distances were analyzed through ANOVA.

The optimal distance between wounds is critical for the wound array model. The wound healing profiles of adjacent wounds, separated by 1, 2, 3, 4, 5, and 6 cm, were monitored over time. The red lines in Fig. 4A define the blood flow signals observed in a 1 cm-thick area from the midline of the inter-wounds beyond the wound edge in four directions. Results are presented as the region of interests (ROI) per unit area at different time points after wounding when compared with ROI per unit area of the respective original skin. The blood flow signals in Fig. 4B reveal that the signal beyond the wound edge was the most outgrown on day 5 after wounding, which regressed on day 7, regardless of the wound distance. On day 5, blood flow signals with a 4 cm distance barely touched each other; therefore, a ≥ 4 cm interval distance between adjacent wounds was required to prevent significant interference during wound healing. In Fig. 4D, blood flow signals in the 1 cm-thick area from the midline of the inter-wound area were analyzed, and the ROI for 1 cm distance of inter-wounds were the highest and subsided along with increased inter-wound distance. The ROI for 4, 5 and 6 cm inter-wound distances reached the background, indicating no interference between adjacent wounds, such that they were close to their ROI of the respective original skin. On the contrary, the ROI for 1, 2 and 3 cm inter-wound distances were significantly higher (P < 0.001) than the background and the ROI for 4, 5 and 6 cm inter-wound distances.

Wound appearances and laser Doppler images from weeks 0 to 7 are shown in Fig. 4C. Quantification of blood flow signals within wound sites reached the highest values at weeks 2 and 3 and subsequently subsided (Fig. 4C,E). In wounds with shorter inter-wound distances, blood flow signals were maintained significantly higher (P < 0.001) than those with longer inter-wound distances above 4 cm. This distinct phenomenon was observed clearly since week 4 and substantiated at least up to 7 weeks. This result indicated interference among wounds with inter-wound distances below 4 cm (Fig. 4E).

Wound closure and contraction were quantified over time (Fig. 4F,G). Wound appearance and wound closure data demonstrated that the wounds were completely closed before week 3 (Fig. 4C,F). Using wound size of 2.5 cm × 2.5 cm, following most reported wound size, inter-wound distance of 1 cm significantly affected the wound closure at day 13–19 (Fig. 4F). This evidences that inter-wound distance indeed affects wound closure, and this will be more significant if larger wound size applies. Nevertheless, there were significant differences in the percentage of wound contraction between inter-wound distances of 1, 2, 3 cm and those of 4, 5, 6 cm (Fig. 4G). This indicates that inter-wound distance lower than 4 cm does affect and reduce the contraction of individual wound due to impedance of adjacent wounds, therefore inter-wound distances ≥ 4 cm are recommended to prevent interference among adjacent wounds.

### A minimal wound size of 4 cm × 4 cm in a wound array model is required to distinguish effects of various treatments

**Figure 5:**
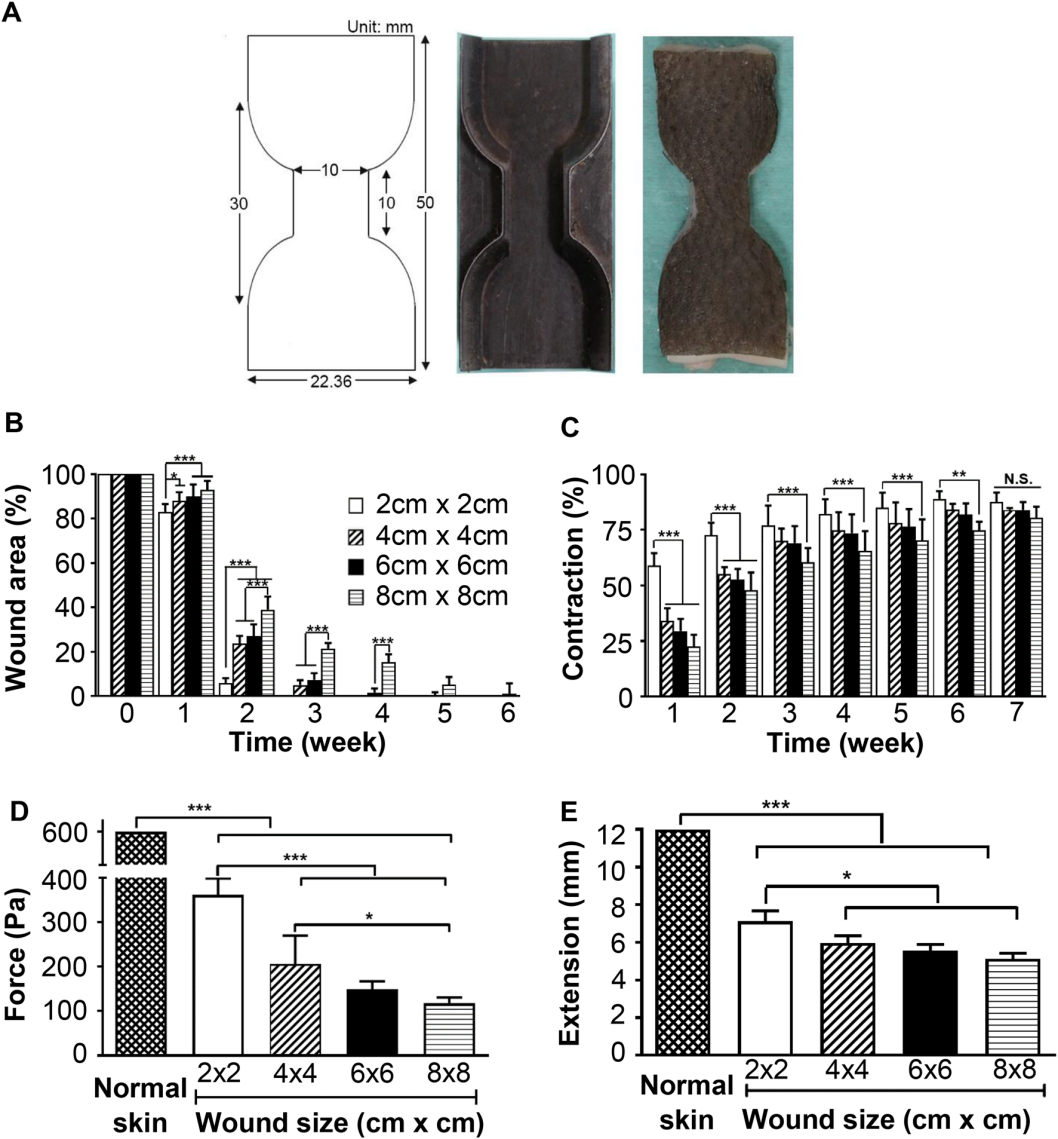
Comparison of various wound sizes for the wound array model in Lanyu pigs. Full-thickness wounds of 2 cm × 2 cm, 4 cm × 4 cm, 6 cm × 6 cm, and 8 cm × 8 cm were created on the dorsal skin of three Lanyu pigs. (**A**) A mould for punching tissues and a punched tissue with a dumbbell shape (10 mm wide in the center). (**B**) Changes in the wound areas with various wound sizes were measured with time. (**C**) Changes in the wound contraction with various wound sizes were measured with time. (**D**) Forces required to lacerate bell-shaped samples of normal skin or regenerated wound tissues were measured using a tensile tester. The samples were collected for measurement at 6 months postoperatively. (**E**) Lengthening of the samples before rupture, as indicated in (**D**) was measured. Statistical significances of each parameter among various wound sizes were analyzed through ANOVA.

In the present study, various wound sizes were created and their effects on the wound healing process were monitored. After statistical analysis, the wound closure rates at wound sizes of 4 cm × 4 cm and 6 cm × 6 cm were not significantly different but were distinct from the wound size of 2 cm × 2 cm and 8 cm × 8 cm (Fig. 5B). The wound healing of the former was definitely too soon to distinguish healing rate of various interventions if any. Therefore, a minimum wound size of 4 cm × 4 cm is suggested. The contraction rate of the wound size of 2 cm × 2 cm was significantly faster than the other wound sizes until week 6, although the percentage of wound contraction was initially inversely related to wound size. Eventually, no significant difference was on wound contraction after week 7 for various wound sizes, approximately 84% of the wound area eventually contracted (Fig. 5C), indicating similar skin structure underneath.

Six months postoperatively, the tensile strengths of groups with wounds measuring 2 cm × 2 cm, 4 cm × 4 cm, 6 cm × 6 cm, and 8 cm × 8 cm were 361 ± 37, 205 ± 64, 149 ± 18, and 117 ± 13 Pa, respectively, which failed to approach the tensile strength of normal skin (Fig. 5D). Furthermore, extension at tearing for the respective groups was significantly lower than that of normal skin, even after 6 months (Fig. 5E).

### Distinctive wound depths affect blood flow signals, wound closure, and wound contraction

**Figure 6:**
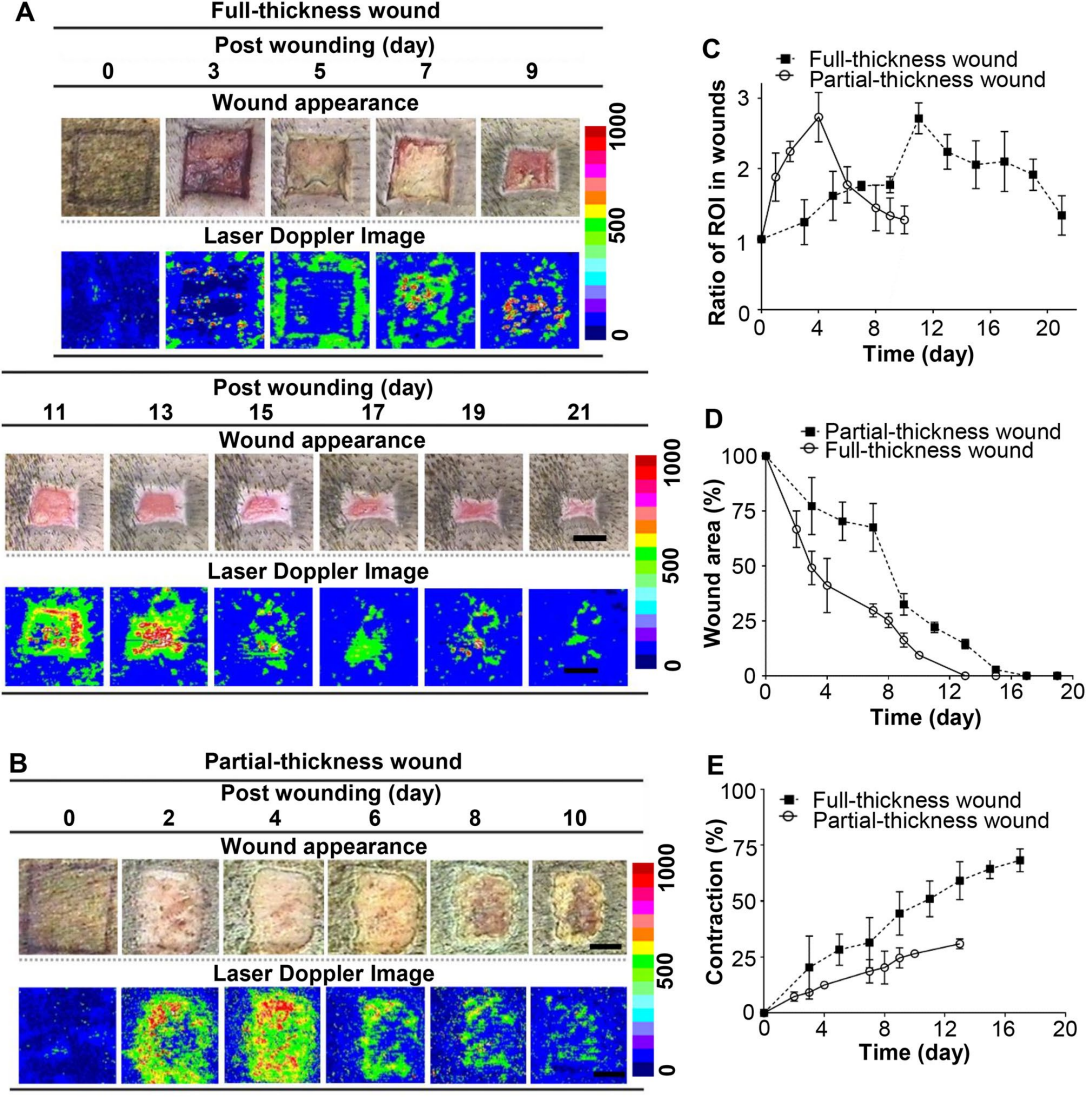
Comparison of partial-thickness (0.23 cm in depth) and full-thickness (0.6 cm in depth) wounds sized 2.5 cm × 2.5 cm on the dorsal skin of Lanyu pigs. Wound appearances and laser Doppler images of (**A**) full- and (**B**) partial-thickness wounds at the indicated time points postoperatively (bar = 1 cm). (**C**) The ratios of ROI of laser Doppler flowmetry were quantified for partial- and full-thickness wounds at indicated time points. (**D**) The percentage of wound area compared with each original wound size was calculated individually for partial- and full-thickness wounds at indicated time points. (**E**) The percentages of wound contraction were calculated for partial- and full-thickness wounds at indicated time points.

We explored the impact of the wound depth on the healing process. Excision wounds of partial-thickness (0.23 cm) and full-thickness (0.6 cm) were created on the dorsal skin surface of Lanyu pigs. Given the limitations of laser Doppler, allowing the acquisition of only surface images, the ratio of blood flow signals peaked 2.73 at day 4 in partial-thickness wounds and 2.71 at day 11 in full-thickness wounds (Fig. 6C). Both partial- and full-thickness wounds showed gradual attenuation of blood flow signals after peaking, approaching signals observed in normal skin on day 0 (Fig. 6A–C).

Figure 6D demonstrates that full-thickness wounds require a longer period for complete closure than partial-thickness wounds, with 8 versus 3 days needed to achieve 50% wound closure. Three distinct phases of wound healing were clearly observed in full-thickness wounds: the inflammatory phase with a slight reduction in wound size, significant wound closure after day 7, and re-epithelialization for wound coverage with a slow slope. Based on changes in the wound margin of full- and partial-thickness wounds, wound contractions of 69.1% and 31.8%, with respective rates of 4.0% and 2.4% per day, were measured at the end of the healing process (Fig. 6E). These findings indicate that wound closure and contraction are closely linked to wound depth.

### Resemblance of skin wounds to normal histological appearance at month 6 post-surgery

**Figure 7:**
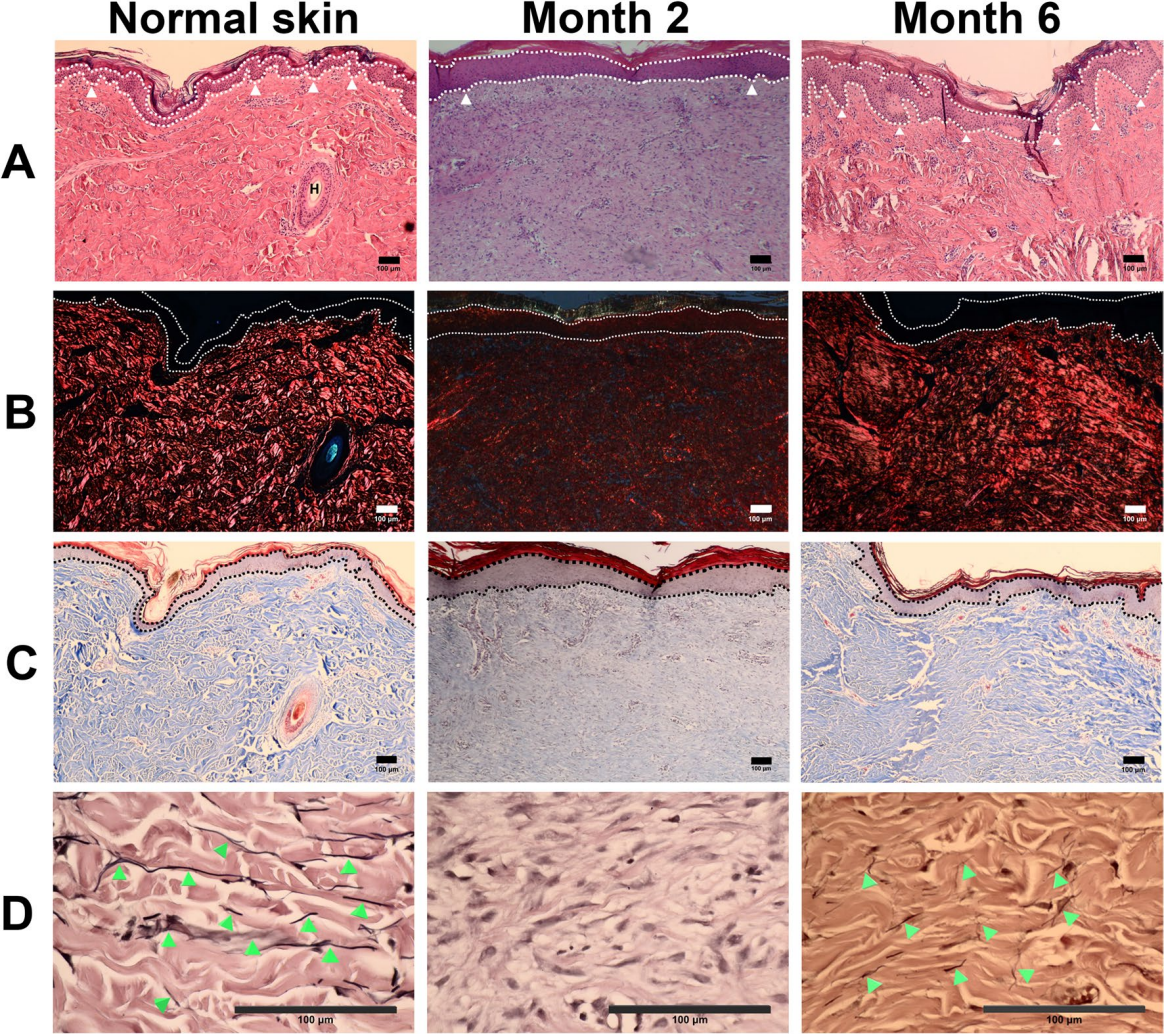
Histological micrographs of normal skin and regenerated skin tissues at months 2 and 6 postoperatively for full-thickness wounds of 2.5 cm × 2.5 cm × 0.6 cm. White dotted lines in (**A**,**B**) and black dotted lines in (**C**) indicate epidermal layers. (**A**) Hematoxylin and eosin-stained micrographs indicate changes in thickness and rete ridges of the epidermis. The rete ridges are indicated by white triangles. The hair follicle is indicated as “H”. (**B**) Picrosirius red staining of polarized micrographs indicates collagen distribution in the dermis. (**C**) Masson’s trichrome staining illustrates collagen biosynthesis in blue color in the dermis. (**D**) Verhoeff’s staining illustrates elastin fibrils in dark brown as indicated by green triangles (bar = 100 µm).

To understand the maturation of repaired tissue, samples were collected at 2- and 6-months post-surgery and stained for histological assessment of collagen deposition and elastin regeneration. At 2 months post-surgery, the repaired tissues showed marked differences from normal skin based on various staining outcomes, including a thicker epidermis, flat rete ridges, the absence of epithelial protrusions extending to the dermis, the absence of distinctly thick collagen fibrils, low collagen content, and low elastin fibril content (Fig. 7). Six months post-surgery, the histological appearance of repaired tissues presented a thicker epidermis and a remark protruding rete ridge structure interlocking with the epidermis and dermis, revealing a transition of epidermal maturation (Fig. 7A). The average thicknesses of epidermal rete ridges were 73 ± 17, 144 ± 18 and 201 ± 73 µm and dermis papillae were 36 ± 6, 90 ± 23 and 70 ± 17 µm, thus the thickness ratios of rete ridges/papillae were 2.0, 1.6 and 2.9 for normal skin, and regenerated skin of months 2 and 6 postoperatively. Compared with normal skin, the puckering of rete ridges reached 95% flatness and 140%, and the collagen contents were 42.5% and 102% at months 2 and 6 postoperatively. In addition, collagen and elastin fibrils were observed in the wound dermis at month 6, along with increased collagen and elastin content and organized architecture of collagen fibrils when compared with samples evaluated at month 2 post-surgery (Fig. 7B,D). Based on the histological results, collagen content in the repaired tissue resembled normal skin 6 months post-surgery.

## Discussion

To establish a reliable skin wound array model, techniques to create wounds, parameters of optimal wound locations, wound sizes, the distance between adjacent wounds, and analysis methods need to be explicitly defined and comprehensively evaluated. Establishing multiple wounds in an array using a singular animal can increase the consistency of results and resolve errors induced by individual animal differences. This can offer researchers a stable platform, with the required reproducibility of wound healing profiles, to reliably screen the effectiveness of various treatments.

Previously^20^, we demonstrated that Lanyu pigs are a more suitable model than Landrace pigs for evaluating the skin wound healing process, owing to their body size and body weight approaching that of humans, with stable wound healing features, especially after puberty. Studies have shown that porcine skin is similar to human skin^15^ in terms of epidermal thickness, subcutaneous composition, and healing processes; this was confirmed in our present study using Lanyu pigs (Fig. 2C–F). Our histological findings demonstrated the homogeneity of the skin structure and thickness at different locations on the dorsum of Lanyu pigs (Fig. 2), confirming its value as an experimental platform for wound healing.

Meeh’s formula^35^: body surface area (BSA) = 8.58 × body weight^2/3^.

According to the above Meeh’s formula, the body surface of a Lanyu pig ranges between 0.73 and 1.46 m^2^ after puberty, weighing between 25 kg at 5 months to 70 kg at 2 years of age. One side of the dorsal skin available for wounds is approximately 4.1–7.7% of the BSA, measuring approximately 25 cm × 12 cm at 5 months and 45 cm × 25 cm at 2 years of age. The wound-array coverage is ~ 1.3 to 2.0% of BSA if wounds measuring 4 cm × 4 cm were created, with 4 cm of distance between adjacent wounds, in which 3 × 2 = 6 and 6 × 3 = 18 wounds could be established for 5-month-old and 2-year-old pigs, respectively. There is no specific restriction for allowable wound area in the Animal Use Protocol only when the study complies with the animal ethical guidelines that animals undergo procedures that cause no pain or distress, or only momentary or slight pain or distress. There are criteria for severity of wound area occupying the whole BSA in humans, that is, < 2% for minor burn, 2–10% for moderate burn, and > 10% for major burn with 3rd degree burn wounds. Accordingly, less than 2% of the whole body surface, which complies with the Animal Protection Act, was used to create an optimized wound-array model.

A limited number of wounds can be created in a mouse, requiring a reference wound for each mouse. For a comprehensive investigation, numerous wounds are necessary when using small animals; the number of animals utilized further increases due to wound numbers and individual differences. Conversely, only a singular large animal might be essential to complete a study, especially for screening or comparing various treatments. As mentioned above, 5–17 treatments can be initially screened and compared with a control open wound, or 1–5 treatments can be screened and compared with an open control in triplicate. Accordingly, one or two Lanyu pigs are sufficient to complete a study when compared with 6–30 mice that may be needed (2 wounds/mouse including a control and treatment; 1–5 treatments; n ≥ 6 for individual variants). In contrast to smaller and limited wound sizes in small animals, larger wounds are possible in larger animals. Moreover, the pig skin system is tight, resembling that of humans, unlike the looser skin system in mice, rats, and rabbits^12^.

Accordingly, the wound array on the dorsum of Lanyu pigs can be used to compare and evaluate various treatments, reducing the number of animals required while minimizing inter-individual variations and further decreasing sample sizes. Therefore, in accordance with the welfare of animals and the 3Rs of replacement, reduction, and refinement^36^ for preclinical trials, this wound array model is crucial for basic and clinical research.

A well-established anesthetic procedure should be employed during surgery, along with proper postoperative care, such as administering antibiotics or analgesia. One side of the dorsal skin is considered at a time to create wounds and is also suggested to allow the pig to lay down on the other dorsal side for rest or sleep. To consider the performance of wound healing, although the thoracolumbar region between the boundaries of both armpits accounts for dorsal skin, some indentations at both ends of approximately 5 cm were considered to prevent skin movement near the front and back legs (Fig. 3A). This also assists in dressing with sterile gauze, elastic bandage, and mesh bandage to prevent them from being stripped off. Large amounts of wound fluid are usually secreted during the initial 7 days, and highly absorbent materials should be applied and changed every other day to cover wounds.

A pig with an array of large wounds could allow more efficient testing and reduce animal usage in the study. However, there are also issues associated with this technique. First, creating a large array of wounds could induce unwanted systemic changes or host responses that would impact wound healing. Second, it would be impossible to characterize the systemic host response to different treatments. Accordingly, mouse models may still be used for initial screening and testing in addition to the availability, cost, and convenience.

Unless a given treatment is particularly relevant to the immune system, which affects the wound healing process, most current therapies and treatments promote skin regeneration; hence, this wound array model is a powerful platform for high-throughput screening of dressings or therapies. In Fig. 4, our results indicate that a distance of at least a 4-cm distance should be maintained between adjacent wounds to prevent wound interaction. This minimum 4-cm distance can be established as the gold standard between adjacent wounds to effectively differentiate various interventions and is first reported in the present study.

Considering the cost and time required for experiments, an optimal wound size of at least 4 cm × 4 cm was suggested to fit multiple wounds in a limited skin area in Lanyu pigs (Fig. 5). Larger wounds may be required to distinguish the efficacy of investigational therapies or dressings. In contrast, smaller-sized wounds may be used, for example, to assess the gene expression of healing profiles, necessitating small amounts of samples. In a study by Elgharably et al.^21^, 32 punch wounds were applied on one side of the pig dorsum with a diameter of 3 mm and a depth of 7 mm. Indeed, each animal study has unique aims and should be uniquely designed to test the theories or clarify specific questions. In other words, a wound size that is optimized for one study may not be ideal for another, depending on the study objectives.

Although complete wound closure was observed after the initial 2 months, the tensile strength of healed skin in the 2 cm × 2 cm group was only approximately 50% of that observed in the normal skin at 6 months post-surgery. Restoration of tensile strength to that of normal skin may require a year or longer, possibly after tissue remodeling. Histologically, the healed matrix at month 6 marginally resembled that of normal skin, as shown in Fig. 7; however, the extent of extracellular matrix remodeling was incomplete.

In order to obtain better blood flow signals and wound closure profiles, different observation time points in Fig. 6A,B were purposely designed. Since there is depth limitation for Laser Doppler imaging and it is elaborate to perform wound observation and images, day 0, 3, and followed with every other day were chosen because of no significant changes in the early time points. However, prominent blood flow changes happened for partial-thickness wounds in the early time, therefore day 0, 1, 2, and followed with every other day were chosen for wound observation and image acquiring. This is not only the reason why distinct pig was chosen for different depth of wound study following 3R principle, but also distinct observation time points could be exemplary for different depth of wound study. The quantitative data of profile comparisons in blood flow, wound closure, and contraction of different depth of wounds are presented in Fig. 6C,D,E, respectively.

Comparing the time points in Fig. 6C,D, we observed that the decreased blood flow signals correlated with wound closure. During the initial 4 days, the wound closure rate in partial-thickness wounds was considerably faster than that in full-thickness wounds (Fig. 6D), correlating with the high blood flow during the granulation phase (Fig. 6C). As the percentage of wound contraction was proportional to time-lapse (Fig. 6E), the second phase of wound closure demonstrated a flat period of the inflammatory phase up to day 7 (Fig. 6D) in full-thickness wounds. After day 7, fibroplasia, extracellular matrix deposition and re-epithelialization by keratinocytes occurred simultaneously. An extraordinarily steep curve of wound closure was observed in the full-thickness group versus a smooth curve in the partial-thickness group. This corresponds to fibroblast-initiated collagen matrix contraction (Fig. 6E). The last phase of wound closure correlated with wound epithelialization, and the slopes were similar in both groups (Fig. 6D).

Ehrlich et al*.*^37^ have reported that re-epithelialization of partial-thickness wounds could be attributed to basal keratinocyte recruitment from wound edges and dermal glands or hair follicles during the early phase of healing, which would stimulate the migration of epithelial cells to form a new epithelial layer. Conversely, cells for re-epithelialization of full-thickness wounds can only be derived from the wound edges, as the dermis has been completely abraded. Therefore, re-epithelialization of partial-thickness wounds is achieved considerably faster than full-thickness wounds. The healing of full-thickness wounds requires dermal regeneration of fibroblasts derived from adipose tissues or wound edges. Berry et al*.*^38^ have reported that fibroblasts are converted to myofibroblasts, and collagen or neosynthesized collagen undergoes contraction; hence, the magnitude of wound contraction in full-thickness wounds was higher than that in partial-thickness wounds (Fig. 6E).

Although there is a common process of wound healing, rate and extent of tissue regeneration in wounds may vary depending on the pig strain, age, wound size, and sampling location within a wound^20^. The thickness of the epidermis and dermis layer, as well as collagen and elastin content, may also differ during any specific stage of wound healing. Nevertheless, qualitative information in Fig. 7 stands for an instruction of the wound healing process. During the transition of epidermal regeneration, the epidermal layer became thicker after wound closure and its basal layer changed from flat to obvious protrusion, finally modified to normal rete ridges. During the process of skin maturity, it was found that the newly formed epidermis and dermal papillae were relatively thicker and flatter than the normal skin and rete ridges developed subsequently. The thickness ratios of rete ridges to dermal papillae turned from 2.0 for normal skin to 1.6 at month 2 and 2.9 at month 6 post-operatively. While the contents of collagen and elastin increased along the process of wound healing, their fibrils developed to the extent in the normal skin. It was found that collagen content was close to normal skin at month 6 postoperatively. The data in Fig. 7 were obtained by sampling from central locations in wounds, indicating that the remodeling phase of wounds was still undergoing at month 6 postoperatively, although wounds closed at approximately 2.5 weeks.

In aggregate, we determined a set of optimal parameters, including the minimal wound size of 4 cm × 4 cm and separation distance of 4 cm, for wound arrays on the dorsal skin, at the 5 cm indentation of the thoracolumbar region between the boundaries of both armpits and the paravertebral region above the rib tips of mature Lanyu minipigs. This model follows the 3R principles and provides a high-throughput platform for precisely screening the effectiveness of different interventional treatments, examining histological changes, and clarifying the underlying molecular mechanisms involved in the wound healing process. This study establishes a golden standard for wound creation and wound care in miniature pigs. In addition, the speed and quality of wound healing may be influenced by the strain of pigs selected, as well as the regeneration capacity of the animals used. In the future, the wound healing efficacy of various biomaterials needs further dedicated investigation.

## Materials and methods

All animal care and surgery protocols were approved by the Livestock Research Laboratory Animal Review Board (IACUC105009), Council of Agriculture, Executive Yuan. All related experiments were performed in accordance with relevant guidelines and regulations. Human skin/tissue samples were not used in the experiments. The only figure of human skin histology was determined the posterior thigh skin of a 33-year-old man, with a registration number of Hu#10. The donor had signed an informed consent with which the subjects’ written consent was approved by the Institutional Research/Human Ethics Committee No. BR-100-102 from the National Cheng Kung University Hospital.

### Animal and anesthesia

Lanyu pigs with the GPI-CRC-PGD homozygous genotype were used in the present study. The Lanyu pigs were obtained from the Taitung Animal Propagation Station of the Livestock Research Institute, Taiwan. The experiments were performed at the Livestock Research Institute, Tainan, Taiwan. All animal care and surgery protocols were approved by the Livestock Research Laboratory Animal Review Board (IACUC105009), Council of Agriculture, Executive Yuan. The pigs were fasted for 24 h before anesthetization prior to surgery. General anesthesia was induced using Zoletil 50 (tiletamine and zolazepam for injection, 2–4 mg/kg; Virbac Laboratories, Carros, France) via intramuscular injection, mixed with atropine (0.05 mg/kg; Astar Pharmaceutical Co., Hsin Chu, Taiwan) and xylazine (2 mg/kg; Lloyd, USA). The anesthetized pigs were placed on the surgical table to create experimental wounds, and inhalational anesthesia was maintained during all surgical procedures using a mixture of 1–3% isoflurane (Panion & BF Biotech Inc., Taiwan) and oxygen.

### Wound creation and postoperative care

After removing the hair using an electrical clipper (Aesculap Favorita 5-GT105, Aesculap Inc., Germany) and razor (Focus R22 Stainless Steel Razor, Focus Inc., Italy), the skin was washed with soap and water. This was followed by drying the body with towels and moving to a surgical table in an aseptic room. Next, the dorsal skin was sterilized sequentially with 7.5% povidone-iodine (Sindine1, Sinphar Pharmaceutical Co., Yilan, Taiwan) and 75% ethanol three times. To ensure that identical wound sizes were created, a transparent film with multiple holes, with designed squares of wound sizes, was placed on the dorsal surface of the pig, and a surgical marker was used to draw precise squares. A scalpel and blade (No.BB536, Aesculap Inc., Germany) were then employed to cut the skin using traced lines, which was followed by using a handmade dermatome (Fig. 1D) to strip the skin and create wounds with the same depth (0.6 cm for full-thickness and 0.23 cm for partial-thickness). After a wound was created, sterile gauze was applied over the wound for hemostasis and cleaning, and the open wound was then covered with Tegaderm dressing (3 M, Taiwan) and an adhesive plaster for protection (Fig. 1E). Next, the wounds were covered with multiple layers of sterile gauze, and an elastic bandage was used to encircle the body a few times to circumscribe wounds from collision, with a mesh bandage placed for further protection (Fig. 1F). As in Fig. 1G, a cloth further covered each pig for final protection.

To clarify the impact of wound location on wound healing, a wound array of 20 wounds, measuring 2.5 cm × 2.5 cm × 0.6 cm, was created on one dorsal side of a Lanyu pig, with 5 cm indentation at both ends of the thoracolumbar region between the boundaries of both armpits. The four full-thickness wounds in the anterior region, closer to the front cranial side, were compared with those in the posterior region, closer to the rear caudal side (n = 4). Five full-thickness wounds in the upper region, closer to the vertebral side, were compared with those in the lower region, closer to the abdomen side (n = 5).

To evaluate the effect of adjacent wound distance on wound healing, multiple full-thickness wounds (2.5 cm × 2.5 cm × 0.6 cm, n = 19) were created on one side of the dorsal skin at inter-wound distances of 1, 2, 3, 4, 5, and 6 cm (Fig. 4A) in one pig and in the opposite direction on the other pig (38 wounds in total). To determine the optimal wound size for the wound array model, full-thickness wounds of 2 cm × 2 cm (n = 22), 4 cm × 4 cm (n = 7), 6 cm × 6 cm (n = 6), and 8 cm × 8 cm (n = 4) were randomly assigned to one side of the dorsal skin in three Lanyu pigs. To evaluate the impact of wound depth on wound healing, a wound array with 24 partial-thickness wounds (2.5 cm × 2.5 cm area) at a depth of 0.23 cm was created on one dorsal side of a Lanyu pig; a wound array with 20 full-thickness wounds (2.5 cm × 2.5 cm area) at a depth of 0.6 cm was created on one dorsal side of another Lanyu pig.

After surgery and recovery from anesthesia, the pigs were returned to their housing, and wounds were monitored every other day. The wounds were also cleaned, and the dressings were changed simultaneously. In addition, weight changes and food intake were monitored daily.

### Tattoo creation and skin growth measurement

Wound closure is influenced by skin expansion during growth. Hence, five tattoos measuring 2.5 cm × 2.5 cm were created at four corners and the center of the dorsal skin in two 5-month-old pigs; the edges of four corners were similar to the four regions as mentioned above. Dorsal skin growth was monitored every other day for 22 days by sketching the size of tattoos and integrating the areas using ImageJ software (National Institutes of Health, Bethesda, MD). Fluctuations in tattooed regions over time were calculated from averages of five tattooed areas at each time point divided by the initial average of five tattooed areas. At the end of the observation period, each tattooed tissue was punched (Dermal Punches, Aesculap Inc., Germany) and collected for histological studies. For the histological assay, the thicknesses of epidermis (*n* = 10 for each tattooed tissue of each pig) and dermis (*n* = 10) were measured and calculated.

### Wound healing assessment

The closure and contraction of wounds are two key parameters for wound healing. Therefore, the apparent changes in wound areas and contraction were monitored over time, as indicated in each experiment. A transparent film was used to mark the edge of each unclosed wound and the margin of each original wound vestige at each indicated time point. The marked transparent films were scanned, and the wound areas were integrated using ImageJ. The changes in the wound area and wound contraction were calculated using the following formulas, using the original wound size at day 0 as the reference.

$${\text{Wound area (}}\% ) = ({\text{wound area remaining/original wound area)}} \times 100 \%$$

$${\text{Wound contraction }}(\%) = ({\text{Original wound area}} - {\text{ Inside area of wound margin at each time point)/original wound area}} \times 100 \% .$$

### Detection of blood flow by laser Doppler image

A laser Doppler flow imaging system (MoorLDI2-HR, Moor Instruments, USA) was used to identify blood flow changes during the wound healing process. Blood flow detection was performed in an operating room at a controlled temperature of 26 °C. The imaging instrument was fixed 42 cm above the anesthetized pigs to scan each wound with the surrounding inter-wound areas. The laser Doppler perfusion index (LDPI), which represents the blood flow at the wound site, was analyzed using Moor LDI v5.3D software (Moor Instruments, Inc., USA). Each wound and its surrounding region (inter-wound area) were scanned using laser Doppler, and the acquired images were stitched into a full-view image. The ratio of regions of interest (ROI) was obtained by comparing the LDPI per unit area of each wound or each 1 cm-thick area from the midline of the inter-wound to that of normal skin using the following formula: ROI = mean of LDPI per unit area in each wound or each 1 cm-thick area from the midline of inter-wound/mean of LDPI per unit area in normal skin.

### Mechanical testing

Six months after surgery, the repaired tissues were punched into dumbbell shapes (10 mm wide) with a narrow parallel-sided portion (22 mm width at ends and 50 mm overall length), centralized to the original wound center, as shown schematically in Fig. 5A. The tensile strengths were measured using a tensiometer (LRX SERIES Materials Testing Machine, SUN/1100 N, AMETEK Inc., USA) to pull each dumbbell-shaped sample with a crosshead speed of 10 mm/min. The strength and energy needed to rupture the dumbbell-shaped samples and the maximum tissue lengthening before rupture were measured and recorded. The force and extension at break were analyzed using NEXYGEN software (Lloyd Instruments Ltd, USA).

### Histological studies

The tissue sections were stained with hematoxylin and eosin, and images were collected using a Panoramic SCAN (3DHISTECH Ltd., Hungary) at the Kaohsiung Chang Gung Memorial Hospital. For different tattoos, variations in the average epidermal and dermal thickness were measured using CaseViewer software version 2.3 (3DHISTECH Ltd. Hungary).

For the histological study of repaired tissues at 2 and 6 months post-wound creation, a scalpel was used to excise a long rectangular piece of tissue from the center of each wound or normal skin. The samples were immersed and fixed in 10% formalin buffer for 24 h, dehydrated, and embedded in paraffin. Samples were sectioned at a 5-µm thickness and stained with hematoxylin and eosin (Merck, Germany) to observe the skin structure; picrosirius red stain (Polysciences Inc., USA) was used to identify the types and distribution of collagen fibrils, Masson’s trichrome stain HT-15 (Sigma-Aldrich Co., Switzerland) was used to determine the architecture and biosynthesis of collagen, and Verhoeff’s stain (Polysciences Inc., USA) was used to clarify the distribution and maturation of elastin fibrils. The stained tissue sections were analyzed under a light microscope (Leica, Germany) or with a polarizer when picrosirius red images were observed.

### Analysis of epidermal thickness, puckering of rete ridges, and collagen content

Using the above histological micrographs, the thickness of the epidermal layer was measured from skin surface to the dermal papillae and epidermal rete ridgeyyy^39^ using ImageJ software by randomly selecting at least 10 fields for each sample. The thickness ratio of the epidermal rete ridge to the dermal papillae was calculated. After sketching the upper and lower contours of the epidermis, the increased puckering ratio of the rete ridges was calculated using the formula: (lower length of healed epidermis/upper length of healed epidermis)/(lower length of normal epidermis/upper length of normal epidermis) × 100%. The collagen content was estimated by quantification of the intensity of picrosirius red staining using ImageJ software.

### Statistical analysis

In the present study, experimental data obtained from measured variables were analyzed using GraphPad Prism v5.0 (GraphPad Software Inc., San Diego, USA). Data are presented as means ± standard deviation (SD). One-way and two-way analysis of variance (ANOVA) as well as student t-test were used as indicated in each figure for statistical analysis among and between groups, and the significances were expressed as follows. *P < 0.05, **P < 0.005, ***P < 0.001.
